# Random Traffic Flow Simulation of Heavy Vehicles Based on R-Vine Copula Model and Improved Latin Hypercube Sampling Method

**DOI:** 10.3390/s23052795

**Published:** 2023-03-03

**Authors:** Hailin Lu, Dongchen Sun, Jing Hao

**Affiliations:** 1 School of Civil Engineering and Architecture, Wuhan Institute of Technology, Wuhan 430074, China; 2 Hubei Provincial Engineering Research Center for Green Civil Engineering Materials and Structures, Wuhan 430073, China

**Keywords:** weigh-in-motion, random traffic flow, correlation, R-vine Copula, Latin hypercube sampling

## Abstract

The rationality of heavy vehicle models is crucial to the structural safety assessment of bridges. To establish a realistic heavy vehicle traffic flow model, this study proposes a heavy vehicle random traffic flow simulation method that fully considers the vehicle weight correlation based on the measured weigh-in-motion data. First, a probability model of the key parameters in the actual traffic flow is established. Then, a random traffic flow simulation of heavy vehicles is realized using the R-vine Copula model and improved Latin hypercube sampling (LHS) method. Finally, the load effect is calculated using a calculation example to explore the necessity of considering the vehicle weight correlation. The results indicate that the vehicle weight of each model is significantly correlated. Compared to the Monte Carlo method, the improved LHS method better considers the correlation between high-dimensional variables. Furthermore, considering the vehicle weight correlation using the R-vine Copula model, the random traffic flow generated by the Monte Carlo sampling method ignores the correlation between parameters, leading to a weaker load effect. Therefore, the improved LHS method is preferred.

## 1. Introduction

Transportation structures such as roads and bridges are designed to carry moving traffic loads. However, with the rapid economic development, the load capacity and occupancy of heavy vehicles are increasing [1,2], generating greater safety hazards to in-service highway bridges and even leading to serious accidents [3]. The heavy vehicle weight parameters indicate strong randomness and significant correlation between parameters; therefore, it is of great importance to fully study the randomness and correlation of heavy vehicle weights and propose a more realistic simulation method for heavy vehicle flow to evaluate the safety of bridge structures.

Several scholars have implemented random traffic flow simulations considering several parameters, such as vehicle type, vehicle weight, axle weight, and vehicle speed, based on data measured utilizing a dynamic weighing system, weigh-in-motion (WIM). For example, Zhouhong et al. [4], Yang et al. [5], and Liang and Xiong [6] developed random traffic flow models applicable to specific regions using Monte Carlo simulation methods. Notably, mass parameters, such as vehicle weight and axle weight, are important for the load effect, and there is a significant correlation between the mass parameters of each traffic model. To build a model closer to a real traffic flow, numerous scholars have used the Copula theory to describe the nonlinear correlation of random parameters. For example, Li et al. [7] analyzed the correlation between vehicle axle weights using the t-Copula function and established a random traffic flow model based on a Monte Carlo simulation. Li [8] analyzed the axle weight correlation according to the Copula distribution function, established a two-dimensional compound Poisson process for vehicle speed and weight using the Levy Copula function, and finally established a random traffic flow model utilizing Monte Carlo simulations. Torres-Alves et al. [9] used the vine Copula model to analyze the axle weight, wheelbase, and vehicle distance to establish a random traffic flow model that considered the correlations through random sampling. Sorianoa et al. [10] used the binary Copula function to construct a joint distribution model for overweight trucks with regard to occupancy and average daily traffic flow. In general, the accuracy of random traffic flow simulations can be improved by considering the correlation between the mass parameters of each vehicle. However, existing simulation methods have the following two shortcomings: First, the parameters of the C-vine and D-vine Copula models used in random traffic flow simulations are all based on fixed-type subjective assumptions, whereas the correlation structure between the variables of each dimension in actual engineering is complex and variable. Furthermore, accurately describing the parameters using a fixed structure is difficult. Therefore, accurately constructing a high-dimensional variable correlation model using the vine Copula model still has certain limitations [11,12]. Second, random traffic flow simulations are mainly conducted by considering the correlation between parameters through the Copula theory and Monte Carlo sampling. The correlation between parameters is difficult to determine using solely the Monte Carlo sampling method because it leads to inaccurate sampled parameters when correlating them. Therefore, a more rational sampling method is urgently needed.

A literature review found the following theories to resolve the above two problems. Morales-Nápoles et al. [13] proposed an R-vine Copula model for topology optimization based on the data-driven nonparametric estimation of the decomposed Copula function, which has better flexibility and practicality. Latin hypercube sampling (LHS), proposed by McKay et al. [14], can achieve stratified sampling to avoid the sampling aggregation phenomenon induced in Monte Carlo sampling while achieving improved accuracy and efficiency. Iman and Conover [15] proposed a simulation method independent of the data distribution. It derives the expected rank correlation matrix using multi-parameter input random variables through matrix transformation to fully preserve the data correlation characteristics. This method can be applied to any type of distribution sampling.

In light of this, 2020 WIM data was collected from the 49,010 Census Station of Interstate 80 in the U.S. to analyze the statistical characteristics of daily traffic flow, vehicle type, vehicle weight, vehicle speed, and other heavy vehicle parameters. Based on this, a scholastic traffic flow model for heavy vehicles was established using the R-vine Copula model with an improved LHS method. The applicability and superiority of the method were verified. This method provides a reference for vehicle load modeling and load design limit optimization.

## 2. Statistical Characterization of Heavy Vehicle Load Parameters

**Figure 1:**
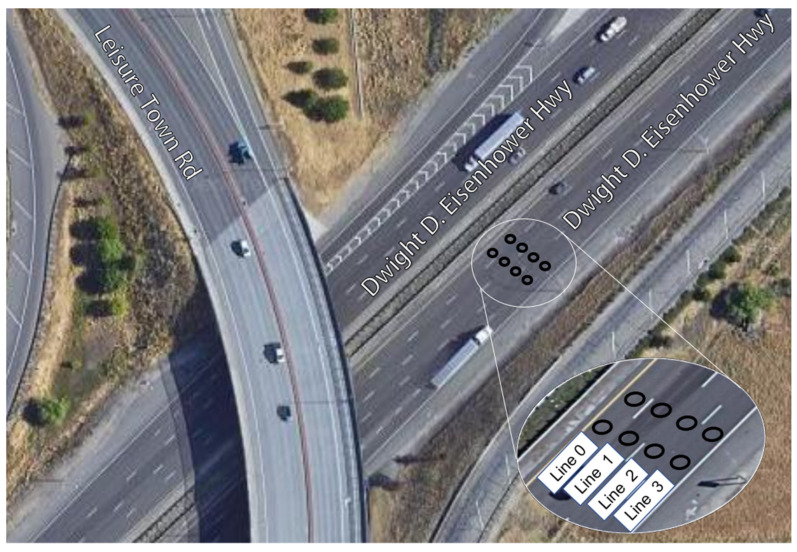
Sensor layout at the mainline traffic survey station.

A WIM system equipped with dual loop sensors was installed on the 49,010 Census Station of Interstate 80, the second largest freeway in Vacaville, California, U.S., as shown in Figure 1. Monitoring data, which includes parameters such as vehicle type, axle weight, vehicle weight, daily traffic volume, vehicle speed, and lane location, was collected, a total of 181,800 data for the year 2020.

### 2.1. Model Classification and Lane Occupancy

According to the classification standard published by the Federal Highway Administration (FHWA) [16], vehicle classification is based on the number of vehicle axles (Axle) and the truck towing method. The towing method is subdivided into single unit (SU), single trailer (ST), and multiple trailer (MT). The vehicle occupancy of each lane at the assessment site is listed in Table 1, and the last six vehicle types in the table are defined by the FHWA as heavy vehicles.

### 2.2. Vehicle Weight Statistics

Parametric and nonparametric methods are commonly used for estimating probability density functions. The parametric method assumes that the random variation of variables conforms to a known distribution and performs parameter estimations based on the monitoring data, whereas the nonparametric method can estimate probability density functions without assuming the type of probability distribution. The parametric method has the following limitations: First, the process of assuming the distribution type is often subjective. Second, the random behavior and dispersion of the monitoring data make it difficult to determine the best assumption of the distribution type. Therefore, based on the monitoring data, the nonparametric kernel density estimation method was used to fit the vehicle weight distribution of heavy vehicles.

Estimating the nonparametric kernel density of the variable *x* as $\hat{f}(x)$, the expression becomes as follows:

(1) $$\hat{f}(x)=\frac{1}{nh}\sum_{i=1}^{n}K(\frac{x-x_{i}}{h}),$$

where *h* is the smooth parameter, *n* is the sample capacity, and *K* is the kernel function. The kernel functions typically used in such applications are the Gaussian, Box, trigonometric, and Epanechnikov.

**Figure 2:**
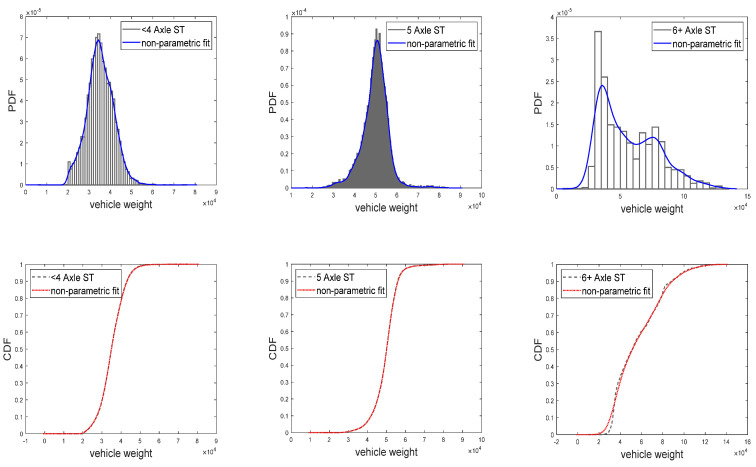

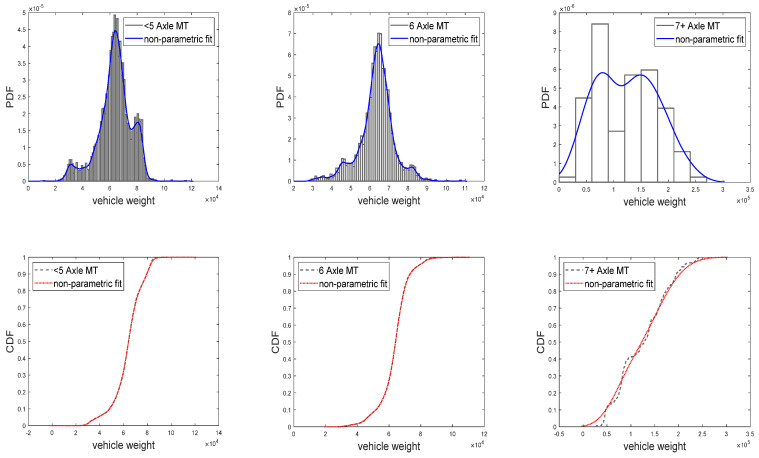
Weight of heavy vehicles.

The nonparametric fit and R^2^ goodness-of-fit tests revealed that the probability density and distribution functions of the weight of the six types of heavy vehicles are well-described by the different kernel functions, as indicated in Figure 2. Among them, the values of the R^2^ goodness-of-fit of the Gaussian kernel density estimation for the weight distribution of the six heavy vehicles were all greater than 0.98. These values were slightly higher than the nonparametric fitting results of the other three kernel functions. In this study, the Gaussian kernel function was adopted, whose expression is as follows:

(2) $$K_{gausssian}=\frac{1}{2\pi }e^{{}^{\left(\frac{-u^{2}}{2}\right)}}.$$

Vehicle weight is the most critical parameter influencing the vehicle load effect. Overloaded heavy vehicles are especially hazardous to the safety of highway bridges. The vehicle weight of each vehicle type in a heavy traffic flow is random, and there is a correlation between the weights of different types of vehicles. To realize an accurate simulation of the heavy vehicle traffic flow, the correlation between the weights of different types of vehicles must be analyzed in addition to considering the random behavior of the weight of each vehicle type. The Pearson correlation coefficient is mainly used to describe a linear correlation applicable to a single-peaked normal distribution. The Kendall rank correlation coefficient can accurately measure the consistency of variation trends and the degree of variation between variables, and is applicable to various distributions. Considering the nonlinear correlation between the vehicle weights of each heavy vehicle type, the Kendall rank correlation coefficient was used as the index to evaluate the correlation. The calculation is expressed as follows:

(3) $$\tau =\sum_{i<k}\left(\mathrm{sign}(x[j]-x[i])\times \mathrm{sign}(y[j]-y[i])\right).$$

The Kendall rank correlation coefficients of heavy vehicle weights are listed in Table 2. These coefficients indicate that the correlation of the vehicle weights of certain types of heavy vehicles is more significant; thus, the correlation needs to be considered when modeling a heavy vehicle traffic flow.

### 2.3. Daily Traffic Statistics

**Figure 3:**
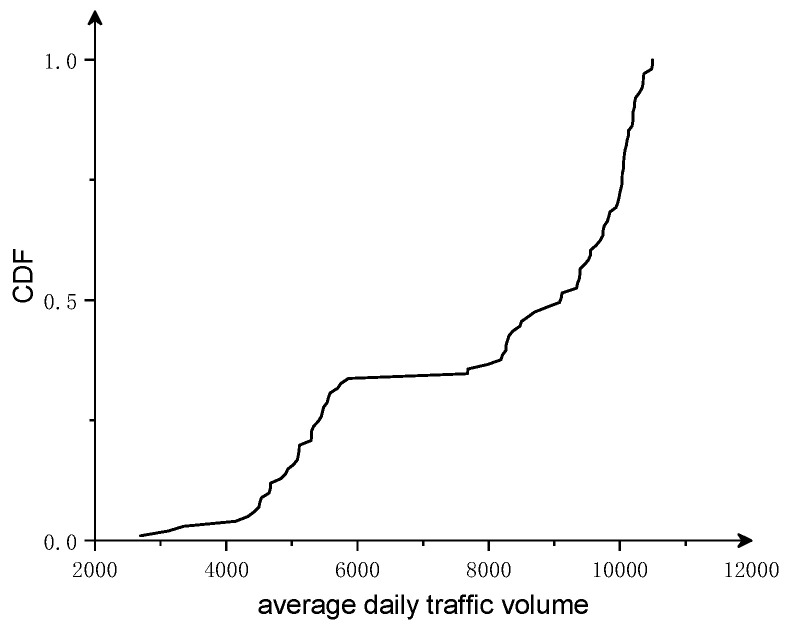
Cumulative distribution of average daily traffic volume.

The cumulative probability density of the daily traffic flow is shown in Figure 3. The daily traffic flow was found to be mainly concentrated from 2500 to 6000 and from 7500 to 11,000 vehicles. When the daily traffic flow is less than 6000 vehicles, it is called the general operation state, and when it exceeds 6000 vehicles, it is called the intensive operation state. The average daily traffic volume is approximately 4900 vehicles/day for the general operation state, 9600 vehicles/day for the intensive operation state, and 7800 vehicles/day for the annual average daily traffic volume, not considering the operation state.

### 2.4. Vehicle Speed Statistics

A statistical analysis of the measured speed for each vehicle in the traffic flow was conducted. The measured speed data of each vehicle was fitted by Gaussian and multi-peaked Gaussian distributions. The goodness-of-fit R^2^ results for each vehicle speed was greater than 0.96. The results indicated that the speed of each vehicle in the traffic flow conformed to Gaussian and multi-peaked Gaussian distributions. The fitting formula is shown in Equation (4), and the fitting parameters of Gaussian and multi-peak Gaussian distributions are listed in Table 3.

(4) $$f(x)=\sum_{1}^{i}a_{i}xe^{-\frac{{\left(x-b_{i}\right)}^{2}}{ci}}.$$

## 3. Six-Dimensional Joint Distribution Model for Heavy Vehicle Weight

### 3.1. Six-Dimensional Joint Distribution Model for Vehicle Weight Based on R-Vine Copula

The Copula theory enables the modeling of joint distributions of multidimensional random variables. Sklar’s theorem [17] provides the relationship between the joint distribution function $F\left(x_{1},x_{2},\cdot \cdot \cdot ,x_{n}\right)$ and the Copula distribution function $C\left(u_{1},u_{2},\cdot \cdot \cdot ,u_{n}\right)$,

(5) $$F\left(x_{1},x_{2},\cdot \cdot \cdot ,x_{n}\right)=C\left(u_{1},u_{2},\cdot \cdot \cdot ,u_{n}\right).$$

By deriving Equation (5), the corresponding probability density function is obtained as follows:

(6) $$f(x_{1},x_{2},\cdot \cdot \cdot ,x_{n})=c(u_{1},u_{2},\cdot \cdot \cdot ,u_{N})\prod_{n=1}^{N}f_{n}(x_{n}).$$

where $c(u_{1},u_{2},\cdot \cdot \cdot ,u_{N})=\frac{\partial C(u_{1},u_{2},\cdot \cdot \cdot ,u_{N})}{\partial u_{1}\partial u_{2}\cdot \cdot \cdot \partial u_{N}}$ is the Copula density function, and N is the density function of the marginal probability density function $f_{n}(x_{n})$ (*n* = 1, 2, ⋅⋅⋅, N).

Although the above Copula model can effectively describe the correlation between random variables, its application to high-dimensional random variables elicits the problems of dimensional disaster and insufficient model accuracy. To resolve these problems, the R-vine Copula model can decompose the high-dimensional Copula function into the product of several two-dimensional Copula functions [18,19]. An n-dimensional R-vine Copula model consists of *n* − 1 layer trees *T*_1_, *T*_2_, …*T_n_*_−1_, where each edge in the tree corresponds to a two-dimensional Copula distribution function, and the set of nodes in the tree is denoted as *N* = {*N*1, *N*2, *N*3, …, *Nn*}. An n-dimensional R-vine structure is subject to the following conditions:

(1) A tree *T*_1_ containing *n* vertices and *n* − 1 edges.
(2) The tree *T_i_* contains *n* − i + 1 vertices and *n* − i edges.
(3) If an edge of the tree *T_i_* connects two nodes, the two edges in the *Ti* − 1 tree corresponding to these two nodes share the same node.

The symbol e represents an edge in the tree and the set of edges E in *E* = (*E*_1, *E*_2, ⋯, *E*_*n*−1); the edge *e* = *a*(*e*), *b*(*e*)|*D*(*e*) of Ei represents *D*(*e*) as a condition of a(e), b(e), and a subset consisting of conditional variables. Each edge *e* = {*a*, *b*} ∈ Ei consists of two nodes connected by an edge *e*. The density function corresponding to edge e is denoted as *Ca*(*e*), *b*(*e*)|*D*(*e*). The n random variables are *X*1, *X*2, …, *X*n, and the subvectors denoted by *XD*(*e*) are determined by the condition set *D*(*e*). The *i* random variables of the marginal probability density function are fi. Based on this, the final joint density function f is shown in Equation (7).

(7) $$f(x_{1},x_{2},\cdot \cdot \cdot x_{n})=\prod_{k-1}^{n}f_{k}(x_{k})\prod_{i=1}^{n-1}\prod_{e\in Ei}c_{a(e),b(e)|D(e)}\left(F(x_{a(e)|D(e)}),F(x_{b(e)|D(e)})\right).$$

Equation (7) shows that once the marginal probability density function $f_{k}(x_{k})$ of the six heavy vehicle weights and the two-dimensional Copula distribution function $c_{a(e),b(e)|D(e)}$ corresponding to each edge in the tree are determined, the joint probability density function $f(x_{1},x_{2},\cdot \cdot \cdot x_{n})$ can be determined, and the initial construction of the six-dimensional joint distribution model of R-vine Copula can also be realized.

### 3.2. Optimization of the Joint Distribution Model of R-Vine Copula

For the six-dimensional joint distribution model of the above six-dimensional R-vine Copula, there exist (6!/2) × 2^(6−2)!/[2(6−4)!]^ possible topologies, and the correlation between the random variables varies with topology. Therefore, determining the best correlation between the random variables becomes a critical problem to resolve for the correlation between high-dimensional random variables. Thus, the joint distribution models of the six heavy vehicle weights were optimized in terms of the connection structure of each layer of the tree, and the joint distribution model as follows: (1) Maximum spanning tree optimization was conducted on the connection structure of each group of trees in the R-vine structure according to the edge weight coefficients. The empirical Kendall weights ${\hat{\tau }}_{ij}$ were used as the evaluation index, and the optimization formula for its structure is as follows:

(8) $$\max\sum_{\mathrm{edges}\;e=\{i,j\}\;\mathrm{in}\;\mathrm{spanning}\;\mathrm{tree}}\left|{\hat{\tau }}_{ij}\right|.$$

(2) To ensure the goodness-of-fit of each marginal distribution model with the final generated joint distribution model, the optimal Copula distribution function was selected using two criteria, namely, the Akaike information criterion (*AIC*) and Bayesian information criterion (*BIC*), to optimize each joint distribution model. The *AIC* and *BIC* were calculated as follows:

(9) AIC=2k−2ln{RVine}(θ|u).

(10) BIC=lnn×k−2ln{RVine}(θ|u|r).

where *k* is the number of parameters, and {*RVine*}(*θ*|*u*|*r*) denotes the set of parameters as *θ*, *u*, and *r*.

In addition, the BIC can solve the problrm that the sample size *n* result to the complex model possesing of large amount of calculation. Moreover, smaller values of AIC and BIC indicate a more accurate description of the correlation between random variables.

**Figure 4:**
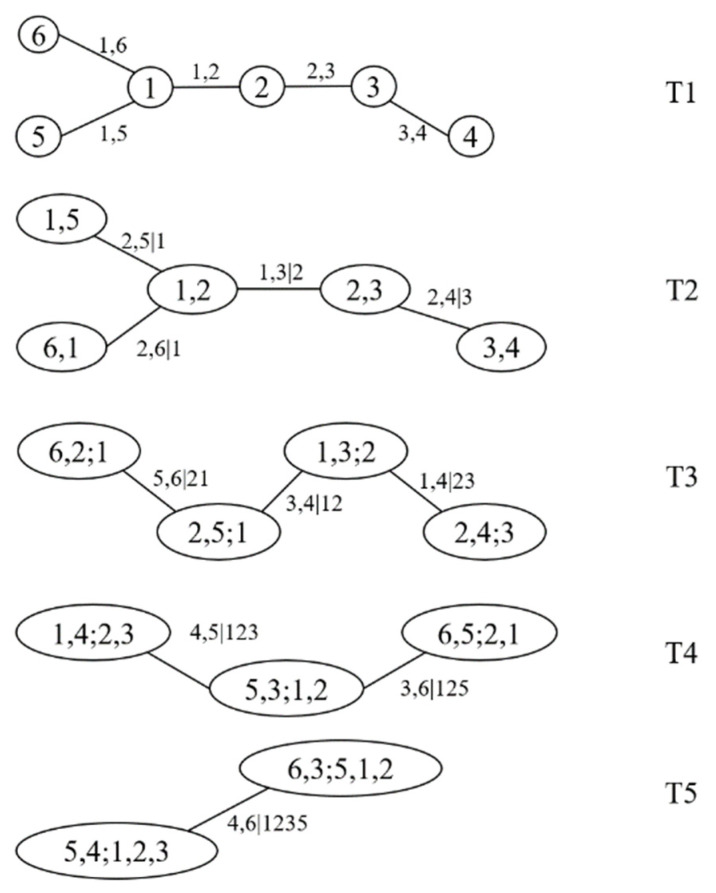
R-vine structure of weight.

The vine structure of the joint distribution model of the six heavy vehicle weights is shown in Figure 4. The marginal distribution models of each layer of the tree, the Copula distribution function, Copula distribution function coefficients (par1 and par2), and AIC and BIC results of each marginal distribution model are listed in Table 4. The AIC and BIC of the joint distribution models of the six heavy vehicles after optimization were −969.2181 and −921.9249, respectively.

## 4. Application of Improved Latin Hypercube Sampling

**Figure 5:**
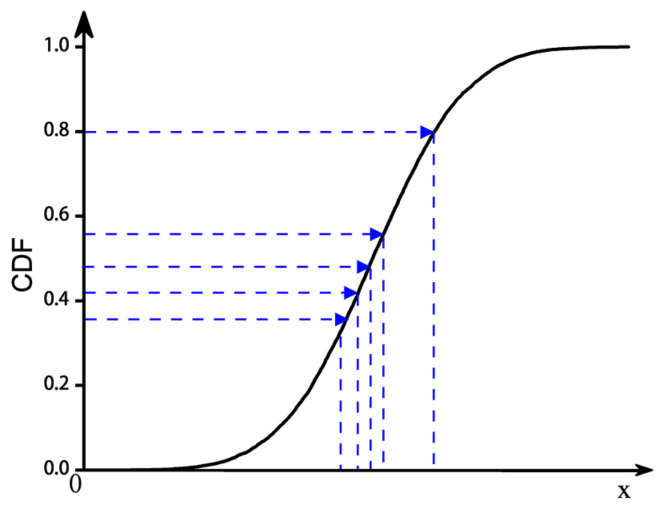
Monte Carlo sampling.

**Figure 6:**
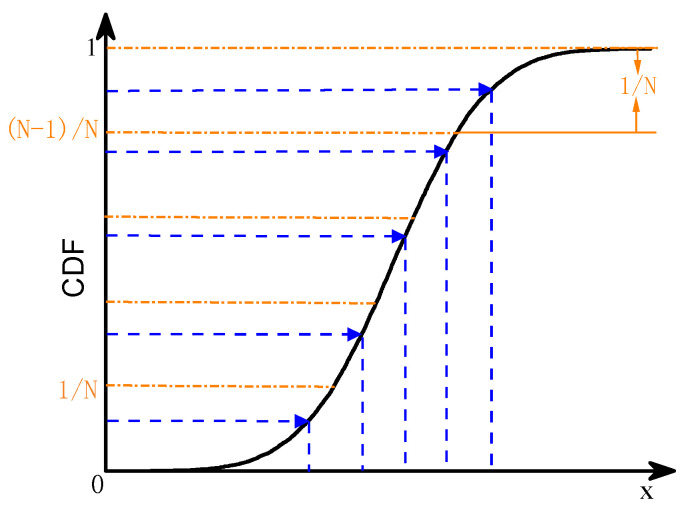
Latin hypercube sampling (LHS).

The Monte Carlo method is often used for sampling in existing random traffic simulations because of its advantages of simplicity and ease of implementation. However, when the number of simulations is small, this method exhibits an aggregation phenomenon, resulting in the neglect of small probability events. Furthermore, this method tends to destroy the correlation between parameters when sampling multidimensional random variables. The LHS method avoids data aggregation by stratifying the probability distribution and is suitable for multidimensional variable sampling with high accuracy and efficiency. Diagrams of the two sampling methods are shown in Figure 5 and Figure 6.

### Fundamentals of Improved Latin Hypercube Sampling

The improved random simulation method proposed by Iman and Conover [15] preserves the correlation between random variables and is applicable to any distribution type. This method is based on the following principle.

If the elements of the random vector x are uncorrelated and there is a correlation matrix I, *C* is the expected correlation matrix generated by transforming *x*. *C* is positive, definite, and symmetric and is equal to the target correlation coefficient matrix C*. According to the Cholesky determinant used by Scheuer and Stoller [20], a lower triangular matrix *P* can be obtained such that *PP*’ = *C*. The desired correlation matrix *C* is obtained by transforming the vector *XP’*. The Cholesky determinant used is as follows:

(11) $$p_{i,i}={\left(c_{i,i}-\sum_{k=1}^{i-1}p_{i,k}^{2}\right)}^{\frac{1}{2}},$$

(12) $$p_{i,j}=(c_{i,j}-\sum_{k=j}^{i-1}p_{i,k}^{}p_{j,k}^{})÷p_{j,j},$$

where *c_i_*_, *i*_ and *p_i_*_, *i*_ are the diagonal elements in the matrix; *i* and *j* represent the rows and columns in the matrix, respectively; and *c_i_*_, *k*_ represents the elements of the *i*-th row and *k*-th column in matrix *C*.

## 5. Simulation of Random Traffic Flow of Heavy Vehicles and Analysis of Load Effect

**Figure 7:**
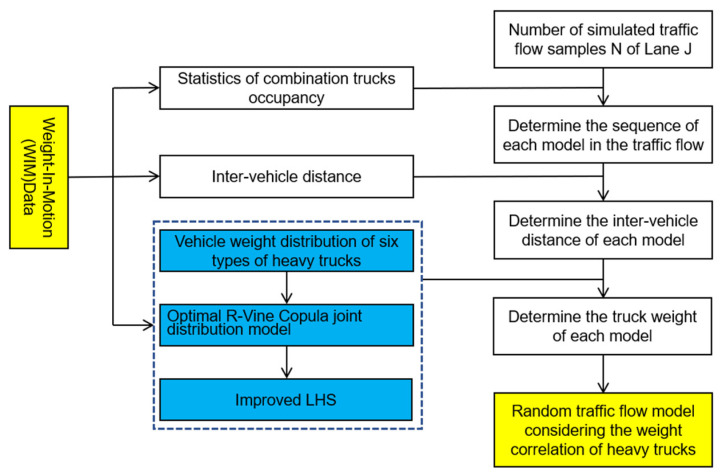
Flow chart of the heavy vehicle random traffic flow model.

Based on the results of the analysis of statistical characteristics of heavy vehicle load parameters, the six-dimensional joint distribution model of vehicle weight, and the improved LHS method mentioned above, the simulation flow chart of the random traffic flow of heavy vehicles is shown in Figure 7. based on the idea of this figure, the simulation program for the random traffic flow of heavy vehicles was prepareand, and this random traffic flow contains 300 vehicles during one hour, which considering the vehicle weight correlation. Notably, the wheelbase-to-axle weight distribution ratios for the six types of heavy vehicles were calculated according to the standard vehicle model provided by the FHWA [21]. Due to sensor performance limitations, the WIM device was not able to collect the following distance during system acquisition, so the authors used the average distance in this article.

The traffic flow samples generated were used with the R-vine Copula model and improved LHS method, called working condition I. To further verify the superiority of this method, working condition II (R-vine Copula model and Monte Carlo sampling method) and working condition III (Monte Carlo sampling method) was also set up, and its samples were calculated separately. The comparison results between the samples generated by the two working conditions and actual model occupancy are listed in Table 5. Working condition I was found to be closer to the monitoring data than working conditions II and III, indicating that the method proposed in this study is superior.

In order to further verify the necessity of considering the correlation of heavy vehicle weight parameters, the load effects of three one-spans simply-supported beams under three working conditions were calculated separately. Firstly, ANSYS, a finite element analysis software, was used to build one-spans of 10 m, 20 m, and 30 m, respectively. The vehicle load samples under the three working conditions were input into the structure to obtain the maximum bending moment of the span section in turn, and the results are shown in Table 6. When the correlation is not considered, the bending moment is the smallest; when the correlation is considered by the R-vine Copula and the Monte Carlo sampling is used, the bending moment is the second largest; when the traffic load sample is obtained by the method of this paper, the bending moment is the largest. This shows that the load effect is conservative if the correlation of the heavy vehicle weight is not fully considered. This is since even if the R-Vine Copula theory is used to consider the vehicle weight correlation, the sampling method still uses Monte Carlo, which leads to the concentration of the sample on the lighter vehicle weight models and, thus, leads to the small load effect results, which is noteworthy.

## 6. Conclusions

In this study, based on the monitored traffic data, a random traffic sample of heavy vehicles considering vehicle weight correlations was generated using the optimal R-vine Copula model and improved LHS method. The following conclusions were obtained.

(1) The nonparametric kernel density estimation can effectively estimate the probability distribution function of the vehicle weight, and there is a correlation between the weight of each type of heavy vehicle. The vehicle speed conforms to the Gaussian and multi-peaked Gaussian distributions.

(2) Various Copula distribution functions of the R-vine Copula model can be selected to connect the marginal distribution functions of each dimension flexibly. Using the maximum spanning tree to choose the optimal topology, the AIC and BIC selected the R-vine Copula model to achieve an accurate description of the joint distribution of the vehicle weight of each vehicle model in the heavy vehicle traffic flow.

(3) The Monte Carlo sampling method destroys the correlation between multidimensional variables, whereas the improved LHS method adequately preserves the data correlation.

(4) The random traffic samples of heavy vehicles generated by considering the vehicle weight correlation based on the optimal R-vine Copula model and improved LHS method are more in line with actual scenarios than other methods. Moreover, the calculated load effect will be smaller if the vehicle weight correlation is not considered.

The authors concluded that the correlation between heavy vehicle weights may be correlated with the industrial distribution and industrialization of each region. Subsequent in-depth exploration of the statistical laws of heavy vehicle weight correlation needs to be investigated based on a large amount of WIM data, using the improved method proposed in this paper, and in conjunction with stochastic process theory.

## Figures and Tables

**Table 1:** Lane occupancy by vehicle type.

Vehicle Type	Lane 0/‱	Lane 1/‱	Lane 2/‱	Lane 3/‱	Total/‱
2 Axle, 4T SU	1790.3000	1788.9550	2333.1040	965.8129	2985.4270
Bus	108.3499	107.5772	56.7047	104.5149	169.5703
2 Axle, 6T SU	3311.0860	3310.1770	3487.1130	2365.9790	5127.4970
3 Axle SU	302.7385	302.6917	418.3685	334.2686	119.7357
4+ Axle SU	33.4853	32.5274	91.9235	18.3512	7.454761
<4 Axle ST	422.6222	421.4048	370.9372	582.9789	140.6703
5 Axle ST	3178.3480	3178.6290	2240.7240	4686.4910	941.3933
6+ Axle ST	19.2045	18.9485	12.2632	29.1005	6.1783
<5 Axle MT	185.4344	185.2683	174.9283	257.5469	44.1158
6 Axle MT	86.8883	86.2973	36.8910	139.6438	27.8277
7+ Axle MT	3.1873	3.1622	3.0405	4.4789	0.6127

**Table 2:** Correlation coefficients of heavy vehicle weight.

	<4 Axle ST	5 Axle ST	6+ Axle ST	<5 Axle MT	6 Axle MT	7+ Axle MT
<4 Axle ST	1	0.3799	0.1725	0.4082	0.4032	0.0886
5 Axle ST	0.3799	1	0.2246	0.3463	0.3101	0.1076
6+ Axle ST	0.1725	0.2246	1	0.27	0.1613	0.194
<5 Axle MT	0.4082	0.3463	0.2700	1	0.4011	0.1313
6 Axle MT	0.4032	0.3101	0.1613	0.4011	1	0.0874
7+ Axle MT	0.0886	0.1076	0.1940	0.1313	0.0874	1

**Table 3:** Vehicle speed fitting parameters for each vehicle type.

Vehicle Type	Parameters		
<4 Axle ST	a = 230.2	b = 58.78	c = 4.506
5 Axle ST	a1 = 1588	b1 = 60.68	c = 2.288
	a2 = 432	b2 = 62.06	c = 5.881
6+ Axle ST	a = 838.8	b = 61.44	c = 3.828
<5 Axle MT	a = 1046	b = 61.2	c = 3.003
6 Axle MT	a1 = 306.4	b1 = 61.44	c1 = 1.296
	a2 = 273.2	b2 = 60.57	c2 = 4.082
7+ Axle MT	a = 188.9	b = 60.58	c = 5.524

**Table 4:** Parameters of R-vine Copula model.

Tree	Edge	Copula	Par1	Par2	AIC	BIC
1	1,6	Frank	−0.7304		0.0157	2.9716
	1,5	Frank	2.1041		−13.9431	−10.9872
	1,2	Clayton	0.3003		−4.8657	−1.9099
	2,3	Rotated Clayton	1.3532		−25.6906	−22.7348
	3,4	Gumbel	1.7000		−900.3170	−897.3612
2	2,5|1	Frank	−0.5347		1.2936	4.2492
	2,6|1	Student	−0.0234		−39.9868	−34.0751
	1,3|2	Frank	−0.0837		1.9577	4.9134
	2,4|3	Rotated Joe	1.0329		1.9902	4.9460
3	5,6|21	Gaussian	0.0137		1.9094	4.8652
	3,4|12	Gumbel	1.0623		2.0451	5.0009
	1,4|23	Frank	0.2530		2.0387	4.9946
4	4,5|123	Frank	−0.6243		0.4744	3.4302
	3,6|125	Frank	−0.2696	5.5349	2.0123	4.9682
5	4,6|1235	Clayton	0.0544		1.8483	4.8041

**Table 5:** Total weight of each vehicle in traffic.

	<4 Axle ST	5 Axle ST	6+ Axle ST	<5 Axle MT	6 Axle MT	7+ Axle MT
Monitoring data	0.0881	0.1329	0.1463	0.1637	0.1614	0.3075
Working condition 1	0.0883	0.1330	0.1463	0.1637	0.1614	0.3074
Working condition 2	0.0905	0.1343	0.1427	0.1682	0.1592	0.3055
Working condition 3	0.0891	0.1348	0.1460	0.1645	0.1609	0.3047

**Table 6:** The total weight share of each model in the traffic flow.

	Working Condition 1/kN·m	Working Condition 2/kN·m	Working Condition 3/kN·m
10 m	3725	3348	3288
20 m	7511	7062	6491
30 m	12,255	11,253	10,776

## Data Availability

The data presented in this study are available on request from the corresponding author. The data are not publicly available due to privacy.
